# Analyzing Smart Healthcare Adoption in Remote-Island Primary Care Clinics: A Hybrid MDM-AHP Study from Kinmen Island

**DOI:** 10.3390/healthcare14030399

**Published:** 2026-02-05

**Authors:** Tsu-Ming Yeh, Hsiao-Yuan Lu, Yi-Hsuan Huang

**Affiliations:** 1 Department of Industrial Engineering and Management, National Quemoy University, Kinmen County 892, Taiwan; 2 Ph.D. Program in Strategy and Development of Cross-Border Industries, National Quemoy University, Kinmen County 892, Taiwan; sport@email.nqu.edu.tw

**Keywords:** smart healthcare, offshore medicine, modified delphi method (MDM), analytic hierarchy process (AHP), key success factors (KSFs)

## Abstract

**Background**: Smart healthcare is increasingly promoted to strengthen primary care services; however, adoption challenges are amplified on remote islands due to geographic isolation and resource constraints. **Objectives**: This study aimed to identify and prioritize key success factors (KSFs) for smart healthcare adoption in remote-island primary care clinics and to examine whether priorities differ across physician subgroups. **Methods**: A hybrid framework combining the Modified Delphi Method (MDM) and the Analytic Hierarchy Process (AHP) was applied. MDM (two rounds) refined a literature-based indicator pool to five dimensions and 20 criteria. AHP pairwise comparisons were collected from 21 physicians in Kinmen to derive weights and rankings. **Results**: System Quality (0.308) was the most critical dimension, followed by Organization (0.221), System Functionality (0.212), Environment (0.165), and Resource Investment (0.094). At the criterion level, Competitive Advantage and Security and Privacy were the two highest-ranked factors, followed by Accuracy and Data Integrity. Subgroup profiles varied across medical specialties and age groups. **Conclusions**: For remote-island primary care, adoption strategies should prioritize system quality and information assurance, while implementation support and resource considerations should be tailored to specialty- and cohort-specific needs.

## 1. Introduction

The United Nations’ World Population Ageing 2020 report indicates that declining fertility rates and increasing life expectancy are accelerating population aging worldwide, thereby placing sustained pressure on healthcare systems [1]. According to population statistics and projections by the National Development Council, Taiwan is expected to become a “super-aged society” by 2025, with people aged 65 or older exceeding 20% of the population [2]. Together with universal National Health Insurance coverage and the growing prevalence of chronic conditions, the demand for healthcare services continues to rise, contributing to escalating medical expenditures [3]. At the same time, shortages in the healthcare workforce have intensified workload and stress among medical personnel, which may ultimately compromise care quality if not addressed effectively [4]. These converging trends underscore the urgency of strengthening primary care capacity and improving service efficiency, particularly in settings where medical resources are already constrained.

In response to these pressures, integrating Artificial Intelligence (AI) and Information and Communication Technology (ICT) into healthcare delivery has been widely regarded as a promising pathway to enhance clinical and administrative efficiency. Smart healthcare solutions can reduce paperwork, streamline clinical workflows, and support healthcare professionals in delivering more timely and higher-quality services [5]. Prior research further suggests that automation and intelligent decision support may help mitigate physician burnout and improve the quality of medical decision-making [6]. In Taiwan, the government has promoted initiatives that accelerate electronic medical record interoperability and the Taiwan Health Cloud project, aiming to strengthen health ICT infrastructure and deliver cloud-based health services at scale [7]. Importantly, as the healthcare sector shifts from disease treatment toward prevention and continuous health management, smart healthcare and mobile/ubiquitous services are increasingly positioned as a key future direction [8].

Primary care clinics play a pivotal role in this transformation because they are the most accessible entry points for the public and can alleviate congestion in large hospitals through bidirectional referral arrangements [9]. However, smart healthcare resources and digital transformation efforts have often been concentrated in large hospitals and urban regions, while rural areas and remote islands commonly face structural disadvantages, including limited specialist availability, constrained budgets, and a persistent digital divide [10]. This imbalance is especially consequential because the cost of inaction in geographically isolated communities can be substantial—delayed diagnosis and fragmented chronic disease management may widen health disparities, increase avoidable referrals, and place additional strain on an already limited acute-care capacity. Moreover, while “telemedicine” can overcome geographic barriers through video consultation, image transmission, and remote monitoring [11], the decision-making logic for adopting smart healthcare in small primary care clinics on remote islands is not necessarily the same as that in hospitals or metropolitan healthcare systems. Indeed, much of the existing literature on smart healthcare adoption has predominantly examined hospital settings or large healthcare organizations, often emphasizing “smart hospitals” or institution-level digital transformation initiatives [12,13]. Consequently, the key success factors (KSFs) for smart healthcare adoption in resource-limited primary care clinics located in remote islands remain insufficiently identified and prioritized from the physicians’ perspective, despite the high practical relevance of this context.

Kinmen provides an appropriate and policy-relevant case for addressing this gap. As a remote-island county, Kinmen has comparatively low medical accessibility, and the island’s acute-care capacity relies heavily on a single hospital [14]. In addition, recent demographic data indicate that Kinmen has a relatively high proportion of older adults (e.g., 65+ population share reported at approximately 19.69% in 2025 [15]), implying a sustained demand for chronic disease care and continuous health management. Although Kinmen lacks medical centers and regional hospitals, the presence of numerous primary care clinics and pharmacies creates an opportunity to develop community-based smart healthcare that shifts the locus of care from hospitals to everyday community settings. Nevertheless, implementing smart healthcare in such constrained environments through a one-size-fits-all strategy is unlikely to succeed. Primary care clinics differ in practice scope, patient profiles, operational capacity, and technology readiness; therefore, adoption priorities may vary not only across institutional levels (primary clinics versus hospitals) but also across medical specialties within the same remote-island context.

Accordingly, this study aims to identify and prioritize the key success factors (KSFs) for smart healthcare adoption in primary care clinics on remote islands, using Kinmen as an empirical case. Specifically, the objectives of this study are threefold: (1) to develop a KSF evaluation framework through the Modified Delphi Method (MDM); (2) to determine the relative importance and ranking of the identified factors using the Analytic Hierarchy Process (AHP) from physicians’ perspectives; and (3) to examine heterogeneity in adoption priorities across physician subgroups (e.g., medical specialties and age groups) and derive corresponding implementation implications for remote-island primary care settings.

## 2. Literature Review

### 2.1. Smart Healthcare

Smart healthcare (often discussed under the broader umbrella of e-Health) refers to the use of Internet and Information and Communication Technologies (ICT) to enhance healthcare services and information delivery [16]. With the rapid evolution of Artificial Intelligence (AI) and the Internet of Things (IoT), smart healthcare has expanded from basic digitalization to the optimization of clinical and service processes through data-driven, connected, and intelligent functions [17]. Shaw et al. [18] further proposed a practical e-Health model that classifies smart healthcare into three domains, namely, m-Health, Interactive Health, and Data-based Health, providing a structured lens for subsequent studies [19].

While the potential benefits of smart healthcare are widely recognized, existing discussions often emphasize technology-driven improvements in efficiency, quality, and accessibility at a general level. From a demand perspective, global aging and the growing burden of chronic disease are key drivers of digital health development [20]. Taiwan faces similar challenges: high chronic disease prevalence among older adults increases the demand for long-term care and continuous management, making technology-enabled monitoring and follow-up increasingly important [21,22]. From a supply-side perspective, Taiwan’s competitive ICT industry and the comprehensive National Health Insurance system provide favorable conditions for smart healthcare development [23]. However, prior work has also noted that implementation and market diffusion remain limited when initiatives are fragmented across isolated projects and lack cross-sector integration, governance alignment, and regulatory readiness [24].

Critical appraisal for remote islands: Much of the smart healthcare discourse implicitly assumes stable digital infrastructure, sufficient implementation capacity, and scale economies (e.g., dedicated IT support, standardized workflows, and organizational slack). These assumptions are less likely to hold in remote-island contexts, where connectivity, staffing, and organizational capacity may be constrained and where clinics may not benefit from the same institutional support available to large hospitals. Moreover, an umbrella review focusing on healthcare professionals reported recurring barriers and facilitators to digital health use (e.g., usability, workflow fit, training, technical support, and infrastructure readiness), suggesting that adoption is often determined by practical implementation conditions rather than technology potential alone [25].

### 2.2. Primary Care

Primary care is widely regarded as the foundation of national health systems. The Institute of Medicine defines primary care as integrated and accessible healthcare delivered by clinicians who address most personal healthcare needs, sustain long-term partnerships with patients, and practice in the context of family and community [26]. In Taiwan, healthcare institutions are categorized into medical centers, regional hospitals, district hospitals, and primary clinics, each playing distinct roles based on capacity and equipment [27]. Primary care clinics are the most accessible frontline units, providing comprehensive and continuous care and serving as the first point of contact for most patients [28].

A well-functioning primary care system is essential for efficient resource allocation across the healthcare system. Prior studies have shown that strengthening primary care can reduce unnecessary hospital visits, improve triage effectiveness, and alleviate emergency department overcrowding, thereby improving overall healthcare system sustainability [29,30]. With the rising prevalence of chronic diseases, primary care clinics are increasingly expected to provide long-term, continuous, and community-based care; for this reason, strengthening primary care has been emphasized as a core strategy for advancing Universal Health Coverage [31].

Critical appraisal for remote islands. Compared with institutions in urban contexts, remote-island primary care clinics often operate under tighter constraints and shoulder a broader range of care responsibilities, including chronic disease follow-up, referrals, and continuity of care when higher-tier services are limited or distant. These characteristics imply that decisions regarding smart healthcare adoption in remote-island primary care clinics may be shaped less by “technology attractiveness” alone and more by feasibility, workflow burden, and risk management (e.g., whether adoption reduces or increases staff workload, whether systems remain reliable under constrained conditions, and whether they align with local practice patterns). A recent scoping review on digital health tools for rural populations with limited access to care highlighted both benefits (e.g., improved access via teleconsultation and remote monitoring) and persistent barriers, such as limited internet connectivity and low familiarity with digital tools, which are directly relevant to remote-island primary care contexts [32].

### 2.3. Decision Criteria

Decision criteria are the factors decision-makers use to evaluate alternatives and determine adoption priorities. To construct an evaluation framework suitable for promoting smart healthcare in primary care clinics on remote islands, this study synthesized evidence from 12 relevant studies and organized commonly used criteria into three broad categories: organization/environment, system/service quality, and resources/functionality.

First, many studies adopt the Technology–Organization–Environment (TOE) framework to examine healthcare IT adoption, emphasizing organizational readiness and external environmental conditions. For example, Dwivedi [33] and Lin et al. [34] used TOE to examine hospital information system integration and cloud care adoption, highlighting organizational capability and environmental support. Nilashi et al. [35] expanded this view by incorporating stakeholders alongside technology, organization, and environment when evaluating hospital information systems. Other studies focusing on telemedicine or cloud services also stress leadership, culture, and planning capability as key determinants of implementation effectiveness [36,37]. Supply-side analyses further demonstrate the importance of leadership support, policy guidelines, and user feedback for smart healthcare development [38].

Second, system quality and service quality are repeatedly identified as core criteria, with an emphasis on reliability, usability, and user experience. Büyüközkan and Çifçi defined e-Health service quality dimensions such as reliability, information quality, and assurance [39]. Studies on m-Health or cloud integration platforms likewise highlight system quality and service quality as essential indicators of adoption success [8,40]. Evaluations of m-Health applications also emphasize compatibility, security, responsiveness, and user satisfaction as important factors [41].

Third, resource investment and functionality requirements are commonly treated as constraints or enabling conditions. Liao and Qiu emphasized service delivery and management issues when evaluating cloud service systems [42]. For telemedicine systems in medically underserved contexts, Su et al. [43] proposed criteria including government regulation, remote technology, system functionality, and clinical feedback. Related studies also show that cost and resource investment are limiting factors in practice [8,38].

Critical appraisal for remote islands (what is less applicable and why): Although TOE-based and quality-centered models provide valuable general insights, they are often developed and validated in hospital or urban settings and may have limited transferability to remote-island primary care clinics for several reasons. (1) Organizational scale and capacity: Many frameworks implicitly assume formalized governance structures and dedicated IT personnel, which small clinics may lack; thus, “organizational readiness” constructs require reinterpretation for micro-organizations. (2) Infrastructure dependence: Quality and functionality criteria may be strongly affected by local connectivity and interoperability constraints, making reliability and data assurance more salient and more difficult to guarantee. (3) Workflow and role multiplicity: Primary care clinics typically operate with lean staffing, and physicians often take on multiple clinical and administrative roles; therefore, adoption criteria should explicitly reflect the time burden, learning costs, and operational simplicity. (4) Contextual heterogeneity: Even within the same remote-island setting, adoption priorities may differ across specialties due to different clinical workflows and service portfolios, implying that a single “best practice” criterion set may be insufficient. Empirically, a national survey of rural public clinics on remote Japanese islands indicated that only about one-quarter of residents used telemedicine and that usage patterns differed by island population size, indicating that feasibility constraints and local conditions can materially shape adoption decisions [44].

Based on the above critique, this study integrated prior criteria while tailoring the framework to the remote-island primary care context. After excluding indicators that appeared only once in the reviewed studies, we identified five main criteria (Table 1): Resource Investment, System Functionality, System Quality, Organization, and Environment.

## 3. Methodology

### 3.1. Problem Definition

Kinmen, as a remote-island county, lacks large medical centers but has numerous primary care clinics that serve as the frontline of community health. Remote-island primary care faces structural challenges such as population aging, limited accessibility to higher-tier medical services, and increasing demand for continuous and high-quality care. Under these constraints, conventional service models may encounter bottlenecks in chronic disease follow-up, referral coordination, and continuity of care. Accordingly, introducing smart healthcare is increasingly viewed as a feasible pathway to strengthen service capability and operational efficiency in remote-island primary care clinics.

Given that decision criteria for smart healthcare adoption may differ across institutional levels and geographic contexts, identifying critical conditions influencing adoption in remote-island primary care clinics remains an important yet insufficiently explored issue. Therefore, this study aims to systematically organize and prioritize key success factors (KSFs) for smart healthcare adoption in remote-island primary care clinics, providing an evidence-based foundation for clinics, system vendors, and policymakers.

### 3.2. Development of the Initial Criteria Pool and Operational Definitions

To construct a context-relevant evaluation framework, we synthesized decision criteria from prior studies on smart healthcare, telemedicine, e-health service quality, and healthcare IT adoption. The initial pool was mapped into five main criteria—Resource Investment, System Functionality, System Quality, Organization, and Environment—to reflect practical decision domains relevant to clinic adoption. Resource Investment refers to the tangible and intangible inputs required for adoption (e.g., cost budgeting, long-term investment planning, and training resources). System Functionality captures whether system features match clinic needs and operational logic (e.g., usability/learnability, compatibility, integration, and security-related functions). System Quality reflects system performance and information reliability (e.g., accuracy, data integrity, timeliness/real-time response, and availability). Organization addresses internal readiness and support conditions (e.g., staff acceptance and capability, management support, vendor support, and infrastructure readiness). Environment represents external conditions shaping feasibility (e.g., policy/regulatory support and external incentives).

### 3.3. Modified Delphi Method (MDM)

This study employed a Modified Delphi Method (MDM) to refine the initial criteria pool and strengthen content validity. The MDM is appropriate for specialized contexts where expert judgment is required to assess the relevance and clarity of candidate indicators through iterative feedback [45,46,47]. A purposive panel of six experts was formed, consisting of three primary care physicians and three university faculty members with relevant expertise in healthcare management and smart healthcare/digital health to balance practical and scholarly perspectives. The experts were invited based on their domain knowledge and their ability to evaluate the feasibility and importance of adoption criteria in resource-constrained primary care settings. Although Delphi panels can be larger, methodological guidance supports the use of smaller panels in specialized domains when the objective is indicator refinement and content validation rather than statistical representativeness [45,46].

The experts rated each indicator using a 5-point Likert scale (1 = very unimportant to 5 = very important). Before the Delphi process began, explicit retention and consensus rules were specified to enhance procedural transparency. Indicators were retained when they demonstrated high perceived importance and acceptable dispersion, based on the following criteria: mean ≥ 4.0, mode ≥ 4, and quartile deviation (QD) ≤ 1 [46,47]. Indicators not meeting these thresholds were reviewed in light of experts’ qualitative feedback and were either revised for clarity or removed when deemed insufficiently relevant to the remote-island primary care context.

Two Delphi rounds were conducted. In Round 1, experts evaluated the initial pool and provided comments on wording and contextual applicability. Two indicators—“Strategic Objective” and “External Competitive Pressure”—received the lowest importance ratings and did not satisfy the predefined retention thresholds; they were therefore removed prior to Round 2. In addition to the quantitative screening, the experts noted that remote-island primary care clinics operate under constrained staffing and relatively stable local demand conditions; as a result, adoption decisions are more strongly shaped by feasibility-related considerations (e.g., reliability, security, workflow fit, and organizational readiness) than by competitive positioning or long-term strategic differentiation. In Round 2, the revised set was re-rated to confirm stability and consensus, and the Delphi process was stopped once all retained indicators met the predefined criteria. The final MDM output yielded five main criteria and the retained sub-criteria, as reported in Table 2. The number of sub-criteria differs across main criteria (e.g., Environment retained two sub-criteria), reflecting expert judgments of contextual salience in remote-island primary care rather than a procedural imbalance.

### 3.4. Analytic Hierarchy Process (AHP)

**Figure 1:**
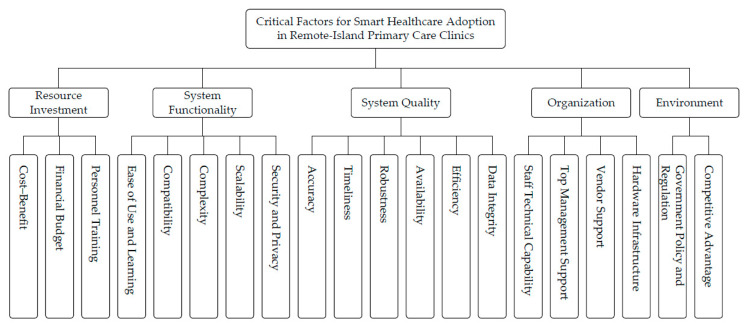
The goal hierarchy structure.

After MDM refinement, the Analytic Hierarchy Process (AHP) was applied to derive the weights and priority rankings of the criteria for smart healthcare adoption in remote-island primary care clinics. The AHP decomposes a complex decision problem into a hierarchical structure and uses structured pairwise comparisons to generate ratio-scale weights [48]. In this study, the hierarchy comprised three levels: the overall goal (prioritizing key success factors for smart healthcare adoption in remote-island primary care clinics), the five main criteria, and the corresponding sub-criteria. As shown in Figure 1, the AHP model was organized to reflect this three-level structure, explicitly linking the study goal to the five criteria and their associated sub-criteria.

The AHP questionnaire employed Saaty’s 1–9 scale, where 1 indicates equal importance and 9 indicates extreme importance of one element over another [48]. Respondents compared (i) the five main criteria and (ii) the sub-criteria within each main criterion. To validate judgment quality, consistency checking was planned as part of the study design. For each comparison matrix, the Consistency Index (CI) and Consistency Ratio (CR) were computed, and responses were considered acceptable when CR < 0.10, consistent with standard AHP guidance [48]. Consistency results are reported in the Section 4.

AHP assumes that criteria are meaningfully distinguishable within the hierarchy and that respondents can provide reasonably consistent judgments. To support these assumptions, indicator definitions were refined through the MDM, and redundancy was minimized during framework construction. As an additional robustness consideration, future research may incorporate sensitivity analyses (e.g., rank stability under small perturbations of weights) to further test the stability of priorities in remote-island primary care contexts.

### 3.5. Data Collection and Participants

The AHP survey targeted physicians practicing in primary care clinics in Kinmen, a remote-island setting in Taiwan. Participants were recruited using a purposive sampling strategy through local professional networks and direct clinic contacts to ensure that respondents possessed relevant clinical experience and contextual knowledge of feasibility constraints in remote-island primary care. The questionnaire was administered following a standardized protocol, including written instructions and examples for pairwise comparisons to enhance response accuracy and reduce interpretation bias.

A total of 26 questionnaires were distributed and all were returned. Prior to analysis, the responses were subjected to eligibility screening and data-quality checks to confirm that AHP comparison matrices could be constructed and that judgments were suitable for weight estimation. Five questionnaires were excluded due to incomplete pairwise comparisons (i.e., missing entries that prevented matrix construction) and/or response patterns that did not support reliable computation (e.g., internally inconsistent or non-computable comparisons identified during the validation procedure). Ultimately, 21 questionnaires were retained as valid, yielding an effective response rate of 80.8%. The final sample included physicians from Western medicine, dentistry, and Chinese medicine, with representation across age groups; detailed participant characteristics are reported in the Section 4. Although the AHP is appropriate for structured expert judgment with modest sample sizes, subgroup analyses may involve small cell sizes; therefore, subgroup findings should be interpreted as indicative and warrant further validation in larger comparative samples.

This study was conducted in accordance with the Declaration of Helsinki and Taiwan’s Human Subjects Research Act. Ethical review and approval were waived because the research involved a non-interventional questionnaire survey of professional personnel (physicians) concerning public policy and management criteria, utilizing anonymous data and posing minimal risk. In accordance with Article 5 of the Human Subjects Research Act and the Ministry of Health and Welfare (MOHW) announcement (Ref No. 1010265075), studies of this nature are exempt from formal institutional review board review. Participation was voluntary, informed consent was obtained prior to questionnaire completion, and no personally identifiable information was collected.

## 4. Research Results

### 4.1. Sample Structure Analysis

A total of 26 questionnaires were distributed, targeting primary care clinic physicians in the Kinmen area, and 26 were returned, resulting in a retrieval rate of 100%. After a rigorous examination of the response content, five invalid questionnaires containing incomplete answers or logical contradictions were excluded. Finally, 21 valid questionnaires were retained, with an effective response rate of 80.8%.

Statistical analysis was conducted on the demographic variables of the valid samples (as shown in Table 3). Regarding gender distribution, 14 physicians were male (accounting for 66.7%) and 7 were female (accounting for 33.3%). In terms of specialty distribution, Western medicine was predominant, with 13 physicians (61.9%), followed by 5 dentists (23.8%) and 3 Chinese medicine physicians (14.3%). The age range of respondents was relatively evenly distributed, mainly consisting of the 41–50 age group and the over 51 group, with 8 physicians each (38.1%), while 5 physicians were aged 31–40 (23.8%). This sample structure indicates that the interviewed physicians mostly possess rich clinical experience, making their opinions valuable for reference.

### 4.2. Consistency Test

The internal consistency of respondents’ pairwise comparisons was determined using the Consistency Ratio (CR) proposed by Saaty [49]. A comparison matrix is considered acceptably consistent when CR ≤ 0.10, indicating that the judgments are sufficiently coherent for deriving reliable priority weights.

Calculations were performed using Expert Choice 2000 software. As shown in Table 4, the CR values for the overall hierarchy and for each criterion-level matrix are all below 0.10, suggesting satisfactory consistency and supporting the credibility of the subsequent AHP-derived weights.

### 4.3. Criterion Weight Results

After aggregating physicians’ judgments using the AHP, the resulting weights for the main criteria and sub-criteria are reported in Table 5. Among the main criteria, System Quality (0.308) receives the greatest weight, followed by Organization (0.221) and System Functionality (0.212), whereas Resource Investment (0.094) ranks last. This ordering suggests that, in remote-island primary care clinics, adoption priorities are primarily shaped by whether the system can provide dependable clinical information support and stable day-to-day operation, rather than by cost considerations alone.

Among the sub-criteria (global weights), Competitive Advantage (0.093) and Security and Privacy (0.091) are the two highest-ranked factors, followed by Accuracy (0.082) and Staff Technical Capability (0.074). Notably, several items share the same global weight (e.g., Compatibility and Scalability, both 0.040); such ties are reported with the same rank in Table 5.

Overall, the pattern indicates that physicians place particular emphasis on information assurance and protection (e.g., security and privacy) and the reliability of core clinical data (e.g., accuracy and integrity), which are especially salient under geographically constrained and resource-limited service conditions.

### 4.4. Group Analysis

Because subgroup sizes are modest (particularly for Chinese medicine), subgroup results are reported as descriptive priority profiles. The comparisons below, therefore, emphasize salient contrasts in weights and ranks rather than statistical significance claims.

#### 4.4.1. Group Analysis by Specialty

Across specialties, the top-level criterion structure shows both common priorities and clear heterogeneity (Table 6). Western medicine and dentistry assign the greatest weight to System Quality (0.266 and 0.397, respectively), whereas Chinese medicine places emphasis on Resource Investment (0.234) and Organization (0.232). The contrast is particularly pronounced for System Quality, which is notably higher in dentistry (0.397) than in Chinese medicine (0.190), indicating that adoption considerations may differ substantially across practice types within the same remote-island environment.

To improve interpretability, Table 6 additionally reports Global Rank within Subgroup (computed as the criterion weight multiplied by the local sub-criterion weight). According to this global view, Western medicine places strong emphasis on external enabling conditions and internal operational readiness (e.g., Government Policy and Regulation and Hardware Infrastructure among the top-ranked items), while dentistry highlights usability and clinical information reliability (e.g., Ease of Use and Learning and Accuracy among the top-ranked items).

In contrast, Chinese medicine assigns comparatively high weight to implementation feasibility in small-clinic operations (e.g., Personnel Training and Financial Budget among the highest-ranked items). These profiles indicate that implementation strategies may need to be differentiated by specialty to address distinct feasibility constraints and operational priorities.

#### 4.4.2. Group Analysis by Age

Priority patterns also differ across age groups (Table 7). At the main-criterion level, the 31–40 age group assigns relatively high weight to Environment (0.315) and Organization (0.298), whereas the 41–50 age group prioritizes System Quality (0.365), and the 51+ age group places the greatest emphasis on System Functionality (0.258). This variability suggests that adoption priorities may shift with practice stage and risk/feasibility concerns.

Global rank within subgroup indicates that the 31–40 age group places the strongest emphasis on external conditions (Government Policy and Regulation and Competitive Advantage are jointly top-ranked), the 41–50 age group focuses on information reliability (Accuracy and Data Integrity among the top-ranked items), and the 51+ age group assigns the highest priority to Security and Privacy. Overall, the age-stratified patterns further indicate that adoption support may need to address different decision focal points across physician cohorts.

### 4.5. Discussion of Research Results

This study provides empirical evidence of how physicians in remote-island primary care clinics prioritize decision criteria when considering smart healthcare adoption. Two findings are particularly noteworthy. First, technological assurance—captured by System Quality—emerges as the dominant consideration, outweighing purely financial concerns such as Resource Investment. Second, the priority structure is not uniform: subgroup analyses reveal meaningful heterogeneity across medical specialties and physician age groups, suggesting that “one-size-fits-all” adoption strategies may be ineffective in constrained primary care settings.

The dominance of System Quality can be interpreted as a rational response to high-stakes clinical work conducted in small organizations with limited operational slack. Unlike larger hospitals that may maintain IT teams, redundancy mechanisms, and formal downtime procedures, small primary care clinics often rely on lean staffing and require physicians to perform multiple roles concurrently. In such settings, instability, latency, or usability frictions can directly disrupt patient flow, increase cognitive burden, and elevate perceived clinical and legal risks. This interpretation is consistent with primary care informatics studies showing that digital tools may fail to support frontline decision-making when workflows are mismatched or when information is incomplete, untimely, or difficult to retrieve [50,51]. It also aligns with the “digital health divide” perspective, which emphasizes that barriers to smart healthcare adoption in underserved areas extend beyond connectivity and include trust-related concerns tied to privacy and secure handling of medical information [52]. In this regard, the high priority assigned to Security and Privacy suggests that adoption in remote islands is not merely an efficiency initiative but also a form of risk management. Practically, vendors and policymakers should treat security assurance as an enabling condition: privacy-by-design features, audit trails, access control, and transparent data governance are likely to reduce perceived risk and strengthen clinician confidence [52].

The prominence of Organization further indicates that smart healthcare adoption in remote-island primary care is fundamentally an organizational change process rather than a simple procurement decision. For small clinics, implementation often entails redesigning day-to-day procedures (e.g., registration, documentation, prescribing, and referrals) and depends heavily on staff competence and willingness to adapt. This is consistent with TOE-based evidence that internal readiness is a key determinant of adoption, especially in settings where resources are limited and change costs are relatively high [35]. Related research on SMEs similarly suggests that smaller organizations face distinctive constraints—limited human resources, limited time for training, and limited capacity to absorb disruption—making organizational and managerial readiness decisive for digital initiatives [53]. Accordingly, clinics may benefit from phased implementation, task-focused training, and clear internal role allocation for troubleshooting and vendor communication. In parallel, vendors can reduce organizational resistance by offering onboarding packages tailored to micro-organizations (e.g., clinic-ready templates, short training modules, and remote support protocols designed for limited staffing). From a policy standpoint, adoption programs should explicitly budget for training time and implementation support, as these are not peripheral costs but major determinants of sustained use.

Differences across age groups suggest that Environment operates as more than a background context in remote-island settings; it can shape the perceived feasibility and timing of adoption. Younger physicians—often in the clinic establishment or expansion phase—may be more sensitive to external resources, policy incentives, and infrastructural enabling conditions. This pattern resonates with the rural telehealth literature emphasizing the role of infrastructure and institutional support, while noting that trust and privacy concerns remain persistent barriers even when connectivity improves [52]. Therefore, infrastructure investment and subsidy programs may be more effective when coupled with governance measures that strengthen trust (e.g., security certification, clear accountability, and data incident protocols), rather than assuming that connectivity improvements alone will ensure adoption.

A particularly original contribution of this study is the specialty-specific heterogeneity in priority profiles. Western medicine and dentistry show a stronger technology orientation and a greater intolerance for technical flaws that disrupt fast-paced workflows, whereas Chinese medicine clinics are more sensitive to resource investment and implementation burden. This can be understood as an issue of product–workflow fit: many commercial smart healthcare solutions are designed around documentation structures and clinical processes typical of Western medicine, and other specialties may face fewer mature, well-aligned options. When workflow fit is weak, clinicians may not perceive sufficient performance gains to justify switching costs, and thus, cost and organizational burden become more salient barriers [50,51]. These findings imply the need for differentiated strategies. Vendors should develop modular and specialty-adaptive systems (e.g., configurable documentation, specialty-specific templates, flexible pricing, and phased adoption packages). Policymakers may also consider specialty-sensitive incentives—such as targeted subsidies or shared-service platforms—to avoid within-island disparities, where only certain specialties can practically adopt and benefit from digital transformation.

From a theoretical standpoint, the results clarify boundary conditions for adoption frameworks in remote-island micro-provider contexts. TOE-based studies in healthcare often highlight organizational readiness and environmental support, while user-level models (e.g., UTAUT) emphasize performance expectancy, effort expectancy, and facilitating conditions [54]. Our findings suggest that, in small primary care clinics under geographic and resource constraints, system quality and data assurance may function as threshold conditions that shape whether other considerations (e.g., cost, policy incentives) can meaningfully influence adoption decisions. Moreover, the observed specialty differences indicate that adoption determinants should incorporate workflow contingencies more explicitly, as the same constructs can carry different operational meanings across specialties within the same constrained geography.

Finally, this study should be interpreted with appropriate caution. Although the sample size reflects the practical constraints of recruiting physicians in remote-island settings, and some subgroup sizes are small, the identified patterns provide informative evidence and generate testable propositions for larger comparative studies across islands and urban settings.

## 5. Conclusions

This study examined smart healthcare adoption priorities in remote-island primary care clinics using Kinmen as an empirical case. By integrating a two-stage Modified Delphi Method with the Analytic Hierarchy Process, we developed a context-specific evaluation framework and derived a physician-informed priority structure. Overall, the findings suggest that adoption in geographically constrained, resource-limited primary care is driven not only by technology availability but also by the need for reliable, secure, and workflow-compatible systems and by the organizational capacity to implement and sustain change in everyday clinical operations. However, the results should be interpreted with appropriate caution. The sample was drawn from Kinmen and reflects the practical constraints of recruiting physicians in remote-island settings; therefore, generalizations to other islands or urban contexts should be made carefully. In addition, while the overall sample size supports the applied AHP procedure, subgroup analyses inevitably involve small group sizes (e.g., Chinese medicine clinics), which limits statistical confidence and calls for further validation. Moreover, this study focused on physicians’ perspectives, but adoption feasibility in primary care may also depend on nurses, administrative staff, and patients, whose viewpoints could provide a more holistic understanding of workflow fit and implementation burden. Finally, the cross-sectional design captures priorities at one point in time; determinants may evolve during and after implementation, which longitudinal research could better capture.

Notwithstanding these limitations, the study yields actionable implications directly derived from the observed priority structure and subgroup patterns. For remote-island primary care, security, privacy protection, and data integrity should be treated as baseline requirements rather than optional enhancements; practical measures such as privacy-by-design functions, transparent data governance, and visible assurance mechanisms (e.g., security certification or compliance checklists) are likely to reduce perceived risk and increase clinician confidence. The subgroup evidence further indicates that implementation and promotion strategies should be more tailored. Vendors can enhance adoption by providing modular and specialty-adaptive systems (e.g., configurable documentation, specialty-specific templates, and phased deployment options) so that clinics can implement functions aligned with their workflows without unnecessary complexity. For specialties that appear more sensitive to investment and implementation burden, flexible pricing (e.g., subscription models) and lightweight onboarding packages may be particularly helpful. Policymakers can also consider tiered support aligned with different needs across physician groups—for example, policy navigation and startup guidance for younger clinic owners, operational stability benchmarking and interoperability support for mid-career physicians, and security assurance resources for senior physicians—thereby improving the feasibility and equity of smart healthcare adoption across clinic types.

From a theoretical perspective, the results also help clarify the boundary conditions of common adoption frameworks in constrained primary care settings: in remote-island micro-providers, system quality and data assurance may function as threshold conditions shaping whether other considerations (e.g., cost incentives or external support) can meaningfully influence adoption decisions, while specialty routines highlight the importance of workflow contingencies within the same geography. Future research should therefore extend this work through comparative designs across other remote islands and urban reference groups, adopt mixed-stakeholder approaches combining physician surveys with clinic staff interviews and patient perspectives, and conduct longitudinal and cost-effectiveness evaluations of specific smart healthcare tools. By bridging these gaps with context-sensitive system design and tiered support, Taiwan can advance more equitable digital health development and strengthen primary care capacity in geographically underserved communities, aligning with broader Universal Health Coverage goals.

## Figures and Tables

**Table 1:** Summary of criteria from the literature.

Goal	Evaluation Items		Sources												Total
	Main Criteria	Sub-Criteria	1	2	3	4	5	6	7	8	9	10	11	12	
Key Factors for Implementing Smart Healthcare in Primary Care Clinics	Resource Investment	Cost–Benefit	ˇ			ˇ		ˇ					ˇ		4
		Financial Budget		ˇ		ˇ	ˇ		ˇ						4
		Personnel Training							ˇ			ˇ	ˇ	ˇ	4
	System Functionality	Ease of Use and Learning			ˇ			ˇ			ˇ				3
		Compatibility	ˇ	ˇ	ˇ	ˇ	ˇ	ˇ	ˇ		ˇ	ˇ		ˇ	10
		Complexity	ˇ			ˇ	ˇ								3
		Scalability			ˇ			ˇ							2
		Security and Privacy	ˇ		ˇ		ˇ	ˇ	ˇ	ˇ	ˇ		ˇ	ˇ	9
	System Quality	Accuracy		ˇ	ˇ					ˇ	ˇ				4
		Timeliness			ˇ				ˇ	ˇ	ˇ		ˇ		5
		Robustness			ˇ			ˇ	ˇ	ˇ			ˇ		5
		Availability			ˇ				ˇ	ˇ	ˇ				4
		Efficiency			ˇ						ˇ				2
		Data Integrity			ˇ					ˇ	ˇ	ˇ			4
	Organization	Staff Technical Capability	ˇ	ˇ			ˇ		ˇ		ˇ		ˇ	ˇ	7
		Top Management Support	ˇ	ˇ		ˇ	ˇ		ˇ		ˇ	ˇ			7
		Strategic Objective		ˇ					ˇ			ˇ			3
		Vendor Support	ˇ	ˇ			ˇ		ˇ		ˇ		ˇ		6
		Hardware Infrastructure	ˇ				ˇ					ˇ	ˇ		4
	Environment	Government Policy and Regulation	ˇ	ˇ			ˇ	ˇ	ˇ				ˇ		6
		External Competitive Pressure	ˇ	ˇ		ˇ	ˇ						ˇ		5
		Competitive Advantage	ˇ			ˇ	ˇ		ˇ						4

1. Dwivedi et al. [33]; 2. Hung et al. [36]; 3. Büyüközkan and Çifçi [39]; 4. Lin et al. [34]; 5. Nilashi et al. [35]; 6. Liao and Qiu [42]; 7. Lin and Chen [38]; 8. Rajak and Shaw [41]; 9. Lotfi et al. [40]; 10. Wolff et al. [37]; 11. Lin [8]; 12. Su et al. [43]. “ˇ” indicates that the corresponding sub-criterion has been explicitly identified or discussed in the referenced literature.

**Table 2:** Statistical results of the second round of the expert questionnaire (MDM).

Main Criteria	Sub-Criteria	Mean	Quartile Deviation	Mode
Resource Investment	Cost–Benefit	4.38	1	4
	Financial Budget	4.13	0.25	4
	Personnel Training	4.38	1	4
System Functionality	Ease of Use and Learning	4.86	0	5
	Compatibility	4.57	1	5
	Complexity	4.43	1	4
	Scalability	4.43	1	4
	Security and Privacy	4.71	0.25	5
System Quality	Accuracy	4.86	0	5
	Timeliness	4.57	1	5
	Robustness	4.57	1	5
	Availability	4.57	1	5
	Efficiency	4.38	1	4
	Data Integrity	4.50	1	5
Organization	Staff Technical Capability	4.13	0.25	4
	Top Management Support	4.50	1	4
	Vendor Support	4.38	1	4
	Hardware Infrastructure	4.25	0.25	4
Environment	Government Policy and Regulation	4.25	0.25	4
	Competitive Advantage	4.13	0	4

**Table 3:** Statistics of retrieved questionnaires.

Variable	Category	Frequency	Percentage
Gender	Male	14	66.7%
	Female	7	33.3%
Specialty	Chinese Medicine	3	14.3%
	Western Medicine	13	61.9%
	Dentistry	5	23.8%
Age	31–40 years old	5	23.8%
	41–50 years old	8	38.1%
	51 years old and above	8	38.1%

**Table 4:** Results of consistency test.

Item	CI	RI	C.R.
Overall Hierarchy	0.02	1.12	0.02
Resource Investment	0.01	0.58	0.02
System Functionality	0.10	1.12	0.09
System Quality	0.06	1.24	0.05
Organization	0.05	0.90	0.06
Environment	0.00	0.00	0.00

**Table 5:** Overall results of criterion weights.

Main Criteria	Weight	Sub-Criteria	Weight	Overall Weight	Rank
A. Resource Investment	0.094	A1. Cost–Benefit	0.372	0.035	16
		A2. Financial Budget	0.227	0.021	19
		A3. Personnel Training	0.401	0.038	14
B. System Functionality	0.212	B1. Ease of Use and Learning	0.113	0.024	18
		B2. Compatibility	0.189	0.040	11
		B3. Complexity	0.078	0.017	20
		B4. Scalability	0.189	0.040	11
		B5. Security and Privacy	0.431	0.091	2
C. System Quality	0.308	C1. Accuracy	0.265	0.082	3
		C2. Timeliness	0.122	0.038	14
		C3. Robustness	0.111	0.034	17
		C4. Availability	0.159	0.049	9
		C5. Efficiency	0.128	0.039	13
		C6. Data Integrity	0.215	0.066	6
D. Organization	0.221	D1. Staff Technical Capability	0.335	0.074	4
		D2. Top Management Support	0.198	0.044	10
		D3. Vendor Support	0.233	0.051	8
		D4. Hardware Infrastructure	0.234	0.052	7
E. Environment	0.165	E1. Government Policy and Regulation	0.437	0.072	5
		E2. Competitive Advantage	0.563	0.093	1

**Table 6:** Results of criterion weights by specialty.

Main Criteria/Sub-Criteria	Western Medicine			Dentistry			Chinese Medicine		
	Weight	Rank	Global Rank	Weight	Rank	Global Rank	Weight	Rank	Global Rank
A. Resource Investment	0.116	5	-	0.109	5	-	0.234	1	-
B. System Functionality	0.212	2	-	0.225	2	-	0.201	3	-
C. System Quality	0.266	1	-	0.397	1	-	0.190	4	-
D. Organization	0.211	3	-	0.153	3	-	0.232	2	-
E. Environment	0.195	4	-	0.116	4	-	0.143	5	-
A1. Cost–Benefit	0.359	2	13	0.143	2	17	0.261	3	7
A2. Financial Budget	0.223	3	18	0.143	2	18	0.328	2	3
A3. Personnel Training	0.418	1	8	0.714	1	3	0.411	1	1
B1. Ease of Use and Learning	0.241	2	7	0.426	1	1	0.134	4	16
B2. Compatibility	0.166	4	17	0.201	3	13	0.196	3	14
B3. Complexity	0.070	5	20	0.035	5	20	0.046	5	19
B4. Scalability	0.186	3	14	0.098	4	16	0.295	2	8
B5. Security and Privacy	0.337	1	3	0.240	2	11	0.329	1	6
C1. Accuracy	0.214	1	6	0.214	1	2	0.030	6	20
C2. Timeliness	0.180	2	9	0.153	5	7	0.059	5	18
C3. Robustness	0.148	5	15	0.157	3	5	0.110	4	17
C4. Availability	0.136	6	16	0.157	3	6	0.172	3	15
C5. Efficiency	0.158	4	12	0.131	6	12	0.248	2	13
C6. Data Integrity	0.164	3	11	0.188	2	4	0.381	1	4
D1. Staff Technical Capability	0.318	2	4	0.291	2	14	0.296	1	5
D2. Top Management Support	0.122	4	19	0.384	1	8	0.246	2	9
D3. Vendor Support	0.209	3	10	0.097	4	19	0.212	4	11
D4. Hardware Infrastructure	0.351	1	2	0.228	3	15	0.246	2	10
E1. Government Policy and Regulation	0.687	1	1	0.500	1	9	0.667	1	2
E2. Competitive Advantage	0.313	2	5	0.500	1	10	0.333	2	12

Note: When multiple items share identical global weights, they are assigned the same rank.

**Table 7:** Results of criterion weights by age group.

Main Criteria/Sub-Criteria	31–40 Years Old			41–50 Years Old			51 Years Old and Above		
	Weight	Rank	Global Rank	Weight	Rank	Global Rank	Weight	Rank	Global Rank
A. Resource Investment	0.077	4	-	0.107	4	-	0.149	5	-
B. System Functionality	0.073	5	-	0.205	3	-	0.258	1	-
C. System Quality	0.237	3	-	0.365	1	-	0.225	2	-
D. Organization	0.298	2	-	0.271	2	-	0.196	3	-
E. Environment	0.315	1	-	0.052	5	-	0.172	4	-
A1. Cost–Benefit	0.100	3	18	0.333	1	11	0.400	1	3
A2. Financial Budget	0.187	2	16	0.333	1	12	0.200	3	15
A3. Personnel Training	0.713	1	5	0.333	1	13	0.400	1	4
B1. Ease of Use and Learning	0.072	4	19	0.151	3	14	0.149	2	12
B2. Compatibility	0.264	2	15	0.142	4	15	0.149	2	13
B3. Complexity	0.148	3	17	0.028	5	20	0.089	5	19
B4. Scalability	0.069	5	20	0.245	2	8	0.105	4	17
B5. Security and Privacy	0.447	1	12	0.433	1	4	0.508	1	1
C1. Accuracy	0.178	3	10	0.287	1	2	0.245	1	7
C2. Timeliness	0.222	1	6	0.044	6	17	0.118	5	18
C3. Robustness	0.133	5	13	0.074	5	16	0.130	4	16
C4. Availability	0.165	4	11	0.226	3	6	0.184	3	11
C5. Efficiency	0.123	6	14	0.111	4	9	0.085	6	20
C6 Data Integrity	0.179	2	9	0.259	2	3	0.238	2	8
D1. Staff Technical Capability	0.479	1	3	0.444	1	1	0.289	1	5
D2. Top Management Support	0.146	3	7	0.317	2	5	0.289	1	6
D3. Vendor Support	0.146	3	8	0.196	3	7	0.176	4	14
D4. Hardware Infrastructure	0.229	2	4	0.044	4	19	0.246	3	9
E1. Government Policy and Regulation	0.500	1	1	0.250	2	18	0.250	2	10
E2. Competitive Advantage	0.500	1	1	0.750	1	10	0.750	1	2

Note: When multiple items share identical global weights, they are assigned the same rank.

## Data Availability

The data presented in this study are not publicly available due to ethical and confidentiality considerations. The questionnaire data were collected from a small sample of physicians in a remote-island setting, and sharing the raw responses could compromise participant anonymity. Aggregated results supporting the findings are provided in the article. Additional information may be made available from the corresponding author upon reasonable request.
